# Correction: The association of mental health with physical activity and its dimensions in Chinese adults: A cross-sectional study

**DOI:** 10.1371/journal.pone.0320346

**Published:** 2025-03-12

**Authors:** Changming Shen, Yan Li

There are errors in the author affiliations. The correct affiliations are as follows:

**1** School of Physical Education, Inner Mongolia University, Yuquan District, Hohhot City, Inner Mongolia Autonomous Region, China, **2** School of Physical Education and Health, Wenzhou University, Chashan Higher Education Park, Wenzhou City, Zhejiang Province, China.
