# Supplementary material for: The association of mental health with physical activity and its dimensions in Chinese adults: A cross-sectional study
Source: PLoS One. 2024 Oct 7;19(10):e0311535. doi: 10.1371/journal.pone.0311535 (PMC11458050; doi:10.1371/journal.pone.0311535)
Supplement: Attachment — Submitted filename: Reviewer.docx [file pone.0311535.s001.docx]

Reviewer #1: 1- The site of data collection is not clear. Did you collect the data online or from the clients to the health centers? Please explain.

Answer: I added: lines 116-117. Data were collected in selected health centers, not online.

2- Did you use string or cluster sampling method in choosing health centers? If yes, you should mention. If not, then please delete this sentence: Due to the use of cluster sampling and a design effect of 1.5 (109-110 lines).

Answer: Yes, it was a cluster sampling. Line: 116

3- Regarding the mental health and physical activity questionnaires, did you measure their reliability with Cronbach's alpha?

Answer: Yes, I had measured it, but because these questionnaires had been used several times before in various Chinese articles, I thought they were highly valid. However, I added Cronbach's coefficient. lines: (138-139 lines and 155-156 lines).

4- There was no difference between female and male in the all variables (p>0.05): You have only mentioned the study of gender differences (157-158 lines). However, in Table 1, there is no data related to each gender and the difference between them. Either add this difference to the table or remove the difference from the text altogether and enter gender as a predictor variable in the regression.

Answer: I added in Table 1

5- There is no need to mention the purpose of the research in the results section (161-164 lines).

Answer: I removed.

Reviewer #2:

1- Abstract: Modify lines 45 to 48 as follows: Notably, the number of walking days per week emerged as the most influential predictor of mental health (β= -0.392), followed by level of weekly physical activity in MET, the number of exercise training sessions per week, weekly exercise training in MET, and weekly walking in MET (β= -0.312, -0.301, -0.212, and -0.202, respectively).

Answer: thanks dear reviewer, I edit. Lines: 45-49

2- Introduction: Provide more detailed reports of the prevalence of mental health problems in China. Furthermore, you have not provided any reports of physical inactivity in China.

Answer: I added in introduction.

3- Methods: The selection model of health centers is a cluster sampling. Please mention it in the sampling section.

Answer: I added.

4- Overall, how many health centers did you choose?

Answer: 33 centers.

5- Did you code or categorize the income level variable? Or have you used the quantitative information of this variable?

Answer: It is specified in Table 1. Annual income (Yuan): <100,000- 100,000 to 300,000- 300,000 to 500,000- 500,000 to 1,000,000- >1,000,000

6- Both questionnaires measure mental health and physical activity well, so they are valid scales. However, did you calculated the reliability of the questionnaires with Cronbach's coefficient?

Answer: Yes, I had measured it, but because these questionnaires had been used several times before in various Chinese articles, I thought they were highly valid. However, I added Cronbach's coefficient. lines: (138-139 lines and 155-156 lines).

7- Please indicate which t-test you used.

Answer: I added in line 160.

8- Results: Please put the results or p value of t-test in table 1.

Answer: I added in Table 1

9- Please remove lines 161-163.

Answer: I removed.

10- Please add Unstandardized coefficients and 95% confidence interval for B in table 2.

Answer: I edit table 2
